# (1*R*,3*R*,3a*S*,8a*R*)-4-Oxo-3-phenyl-1-[(1*R*)-1-phenyl­eth­yl]deca­hydro­cyclo­hepta­[*b*]pyrrol-1-ium bromide

**DOI:** 10.1107/S1600536812028073

**Published:** 2012-06-27

**Authors:** Victor B. Rybakov, Dmitry S. Belov, Evgeny R. Lukyanenko, Alexander V. Kurkin, Marina A. Yurovskaya

**Affiliations:** a Department of Chemistry, Moscow State University, 119992 Moscow, Russian Federation

## Abstract

The title chiral compound, C_23_H_28_NO^+^·Br^−^, was obtained from an optically active amino­ethanol precursor. The pyrrolidine heterocycle has an envelope conformation, with the C atom α-positioned with respect to the keto group deviating by 0.570 (6) Å from the mean plane of other atoms. The *trans*-fused seven-membered ring adopts a pseudo-chair conformation. The two phenyl rings form a dihedral angle of 85.1 (2)°. The cationic center and the bromide anion are connected through an N—H⋯Br hydrogen bond.

## Related literature

For general background to the aza-Cope–Mannich sequence, see: Overman (1992 ▶, 2009 ▶). For natural products with cyclo­hepta­[*b*]pyrrolidine, see: Earley *et al.* (2005 ▶); Martin *et al.* (2008 ▶). For biologically active compounds, see: Tamiz *et al.* (2000 ▶). For the preparation of *cis*-cyclo­hepta­[*b*]pyrrolidines, see: Belov *et al.* (2011 ▶). For standard bond lengths, see: Allen *et al.* (1987 ▶).
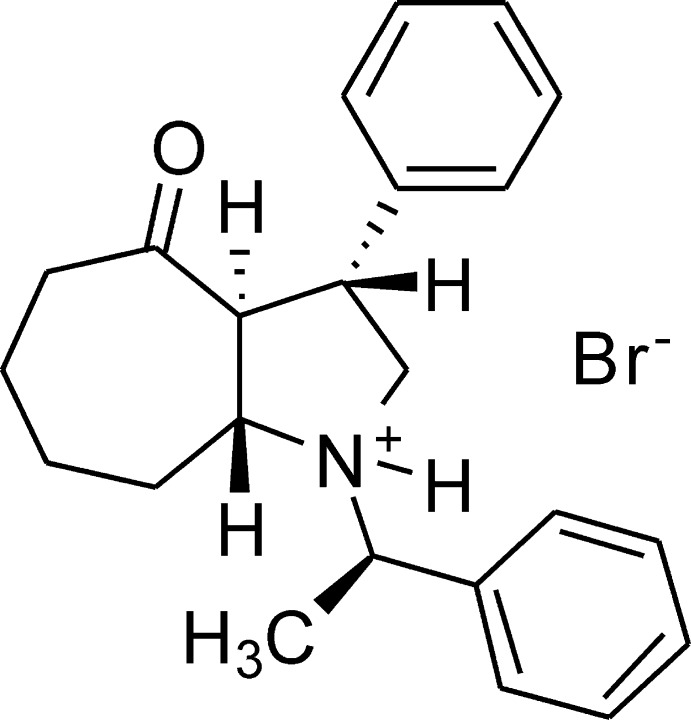


## Experimental

### 

#### Crystal data

C_23_H_28_NO^+^·Br^−^
*M* *_r_* = 414.36
Monoclinic, 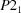

*a* = 6.7996 (4) Å
*b* = 13.3136 (8) Å
*c* = 11.3167 (8) Å
β = 94.449 (5)°
*V* = 1021.38 (11) Å^3^
*Z* = 2
Mo *K*α radiation
μ = 2.02 mm^−1^
*T* = 295 K
0.25 × 0.25 × 0.13 mm

#### Data collection

Stoe STADI-VARI Pilatus-100K diffractometer
Absorption correction: integration (*X-RED32*; Stoe & Cie, 2012 ▶) *T*_min_ = 0.229, *T*_max_ = 0.482
8956 measured reflections
2995 independent reflections
2116 reflections with *I* > 2σ(*I*)
*R*_int_ = 0.071

#### Refinement

*R*[*F* ^2^ > 2σ(*F* ^2^)] = 0.043
*wR*(*F* ^2^) = 0.104
*S* = 0.97
2995 reflections
230 parameters
1 restraint
H-atom parameters constrained
Δρ_max_ = 0.41 e Å^−3^
Δρ_min_ = −0.29 e Å^−3^
Absolute structure: Flack (1983 ▶), 897 Friedel pairs
Flack parameter: −0.018 (14)

### 

Data collection: *X-AREA* (Stoe & Cie, 2012 ▶); cell refinement: *X-AREA*; data reduction: *X-RED32* (Stoe & Cie, 2012 ▶); program(s) used to solve structure: *SHELXS97* (Sheldrick, 2008 ▶); program(s) used to refine structure: *SHELXL97* (Sheldrick, 2008 ▶); molecular graphics: *ORTEP-3* (Farrugia, 1997 ▶); software used to prepare material for publication: *WinGX* (Farrugia, 1999 ▶).

## Supplementary Material

Crystal structure: contains datablock(s) global. DOI: 10.1107/S1600536812028073/ld2065sup1.cif

Additional supplementary materials:  crystallographic information; 3D view; checkCIF report

## Figures and Tables

**Table 1:** Hydrogen-bond geometry (Å, °)

*D*—H⋯*A*	*D*—H	H⋯*A*	*D*⋯*A*	*D*—H⋯*A*
N1—H1⋯Br1	0.91	2.44	3.266 (4)	151

**Fig. 1.**
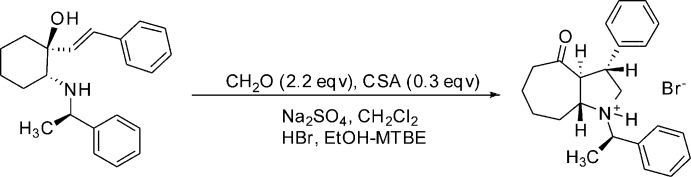
Synthetic path for the title compound.

**Fig. 2.**
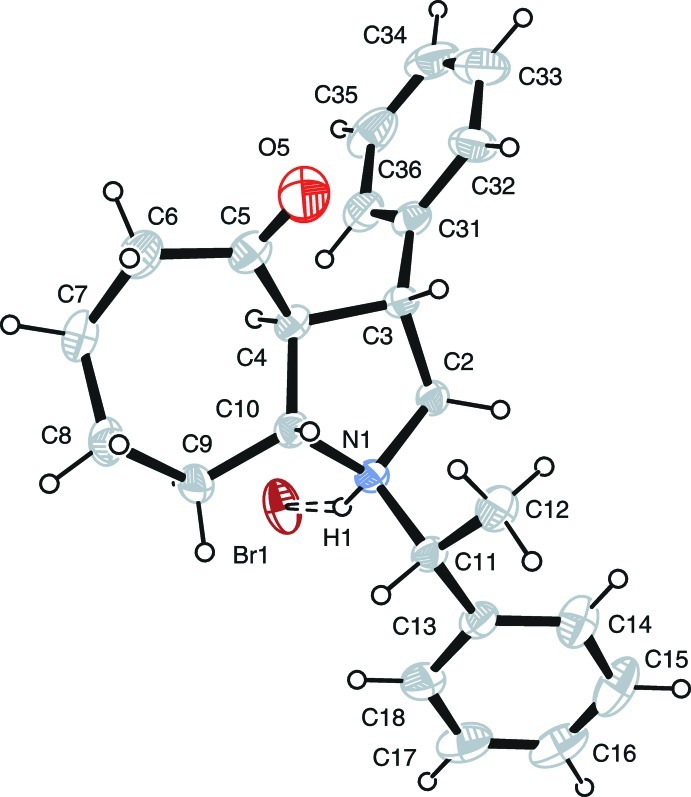
The molecular structure of the title compound with the atom numbering scheme. Displacement ellipoids are drawn at the 30% probability level. H atoms are presented as small spheres of an arbitrary radius.

## supplementary crystallographic information

### Comment

Cyclohepta[*b*]pyrrolidine moiety has been found in several natural products - gelsemine (Earley *et al.*, 2005), actinophyllic acid (Martin *et al.*, 2008) and other biologically active compounds (Tamiz *et al.*, 2000). For natural products and pharmaceuticals containing more than one chiral center, identification of diastereomers is of great importance because of their different physical and, most importantly, biological properties. Recently we reported an improved procedure for preparation of *cis*-cyclohepta[*b*]pyrrolidines (Belov *et al.*, 2011). In this article we developed a method for stereoselective synthesis of the *trans*-cyclohepta[*b*]pyrrol core *via* aza-Cope-Mannich sequence (Overman, 1992; 2009) in an optically pure form using (1*R*)-1-phenylethanamine as a chiral auxillary (Fig. 1). The molecular structure is presented in Fig. 2. All bond lengths are within expected ranges (Allen *et al.*, 1987).

### Experimental

To a vigorously stirred mixture containing (1*S*,2*R*)-2-{[(1*R*)-1-phenylethyl]amino}-1- [(*E*)-2-phenylethenyl]cyclohexanol (1.00 g, 3.1 mmol), anhydrous Na_2_SO_4_ (3.10 g, 21.7 mmol, 7 eqv), camphorsulfonic acid (0.22, 0.9 mmol, 0.3 eqv) and CH_2_Cl_2_ (16 ml), 98 ml of formalin (37% in water, 0.54 ml, 6.8 mmol, 2.2 eqv) were added dropwise at RT. The reaction mixture was vigorously stirred overnight. The mixture was washed with saturated NaHCO_3_ solution (50 ml) and dried (Na_2_SO_4_). Concentration gave product which was dissolved in *MTBE*-*Et*OH (1:1, 10 ml) and aqueous HBr was added to the solution (0.35 ml) causing precipitation of (3*R*,3*aS*,8*aR*)-3-phenyl-1-[(1*S*)-1-phenylethyl] octahydrocyclohepta[*b*]pyrrol-4(1*H*)-one as a hydrobromide salt (1.16 g, 90%). *M*.p. = 498.1–498.5 K.

### Refinement

The N- and C-bound H atoms were placed in calculated positions with C—H 0.93 Å–0.98 Å and N—H 0.91 Å and refined as riding with *U*_iso_(H) = 1.2 and (1.5)*U*_eq_(C,*N*). The positions of H atoms of methyl group were rotationally optimized by using instruction HFIX 137 in *SHELXL* program.

### Crystal data

**Table:**

C_23_H_28_NO^+^·Br^−^	*F*(000) = 432
*M**_r_* = 414.36	*D*_x_ = 1.347 Mg m^−^^3^
Monoclinic, *P*2_1_	Melting point = 498.1–498.5 K
Hall symbol: P 2yb	Mo *K*α radiation, λ = 0.71073 Å
*a* = 6.7996 (4) Å	Cell parameters from 6482 reflections
*b* = 13.3136 (8) Å	θ = 1.8–29.2°
*c* = 11.3167 (8) Å	µ = 2.02 mm^−^^1^
β = 94.449 (5)°	*T* = 295 K
*V* = 1021.38 (11) Å^3^	Prism, colourless
*Z* = 2	0.25 × 0.25 × 0.13 mm

### Data collection

**Table:**

Stoe STADI-VARI Pilatus-100K diffractometer	2995 independent reflections
Radiation source: LFF Sealed Tube	2116 reflections with *I* > 2σ(*I*)
Plane graphite monochromator	*R*_int_ = 0.071
Detector resolution: 5.81 pixels mm^-1^	θ_max_ = 26.0°, θ_min_ = 1.8°
Rotation method scans	*h* = −8→8
Absorption correction: integration (*X-AREA*; Stoe & Cie, 2012)	*k* = −16→11
*T*_min_ = 0.229, *T*_max_ = 0.482	*l* = −13→9
8956 measured reflections	

### Refinement

**Table:**

Refinement on *F*^2^	Secondary atom site location: difference Fourier map
Least-squares matrix: full	Hydrogen site location: inferred from neighbouring sites
*R*[*F*^2^ > 2σ(*F*^2^)] = 0.043	H-atom parameters constrained
*wR*(*F*^2^) = 0.104	*w* = 1/[σ^2^(*F*_o_^2^) + (0.0568*P*)^2^] where *P* = (*F*_o_^2^ + 2*F*_c_^2^)/3
*S* = 0.97	(Δ/σ)_max_ < 0.001
2995 reflections	Δρ_max_ = 0.41 e Å^−^^3^
230 parameters	Δρ_min_ = −0.29 e Å^−^^3^
1 restraint	Absolute structure: Flack (1983), 897 Friedel pairs
Primary atom site location: structure-invariant direct methods	Flack parameter: −0.018 (14)

### Special details

**Table:**

Geometry. All s.u.'s (except the s.u. in the dihedral angle between two l.s. planes) are estimated using the full covariance matrix. The cell s.u.'s are taken into account individually in the estimation of s.u.'s in distances, angles and torsion angles; correlations between s.u.'s in cell parameters are only used when they are defined by crystal symmetry. An approximate (isotropic) treatment of cell s.u.'s is used for estimating s.u.'s involving l.s. planes.
Refinement. Refinement of F^2^ against ALL reflections. The weighted R-factor wR and goodness of fit S/i> are based on F^2^, conventional R-factors R are based on F, with F set to zero for negative F^2^. The threshold expression of F^2^ > 2σ(F^2^) is used only for calculating R-factors(gt) etc. and is not relevant to the choice of reflections for refinement. R-factors based on F^2^ are statistically about twice as large as those based on F, and R-factors based on ALL data will be even larger.

### Fractional atomic coordinates and isotropic or equivalent isotropic displacement parameters (Å^2^)

**Table:**

	*x*	*y*	*z*	*U*_iso_*/*U*_eq_	
Br1	−0.23660 (6)	0.27140 (5)	−0.02750 (6)	0.0688 (2)	
N1	0.1339 (5)	0.1146 (3)	−0.0108 (3)	0.0368 (8)	
H1	0.0142	0.1407	−0.0358	0.044*	
C2	0.2616 (6)	0.2019 (4)	0.0343 (4)	0.0395 (10)	
H2A	0.1855	0.2636	0.0320	0.047*	
H2B	0.3723	0.2105	−0.0139	0.047*	
C3	0.3348 (4)	0.1755 (3)	0.1631 (2)	0.0388 (10)	
H3	0.4556	0.1353	0.1630	0.047*	
C31	0.3719 (4)	0.2642 (3)	0.2411 (2)	0.0458 (9)	
C32	0.5629 (4)	0.2789 (3)	0.2920 (2)	0.0660 (14)	
H32	0.6633	0.2354	0.2738	0.079*	
C33	0.6034 (13)	0.3576 (6)	0.3693 (6)	0.086 (2)	
H33	0.7321	0.3668	0.4016	0.104*	
C34	0.4647 (15)	0.4206 (7)	0.3991 (6)	0.091 (3)	
H34	0.4953	0.4724	0.4525	0.110*	
C35	0.2722 (14)	0.4082 (5)	0.3494 (6)	0.083 (2)	
H35	0.1731	0.4518	0.3692	0.100*	
C36	0.2295 (9)	0.3297 (4)	0.2695 (5)	0.0582 (14)	
H36	0.1016	0.3221	0.2353	0.070*	
C4	0.1632 (7)	0.1082 (4)	0.2013 (4)	0.0406 (10)	
H4	0.0528	0.1510	0.2208	0.049*	
C5	0.2251 (8)	0.0412 (4)	0.3074 (5)	0.0548 (13)	
O5	0.3950 (7)	0.0369 (5)	0.3429 (4)	0.0983 (18)	
C6	0.0720 (9)	−0.0179 (6)	0.3648 (5)	0.0705 (16)	
H6A	0.0666	0.0073	0.4450	0.085*	
H6B	0.1173	−0.0869	0.3711	0.085*	
C7	−0.1365 (8)	−0.0190 (5)	0.3073 (5)	0.0598 (14)	
H7A	−0.1853	0.0494	0.3033	0.072*	
H7B	−0.2188	−0.0566	0.3579	0.072*	
C8	−0.1601 (10)	−0.0640 (5)	0.1835 (6)	0.0589 (16)	
H8A	−0.2954	−0.0866	0.1682	0.071*	
H8B	−0.0757	−0.1226	0.1815	0.071*	
C9	−0.1110 (7)	0.0073 (4)	0.0835 (5)	0.0496 (12)	
H9A	−0.1374	−0.0270	0.0083	0.059*	
H9B	−0.1983	0.0649	0.0836	0.059*	
C10	0.1002 (6)	0.0443 (4)	0.0921 (4)	0.0402 (11)	
H10	0.1891	−0.0135	0.0893	0.048*	
C11	0.2090 (7)	0.0586 (4)	−0.1164 (4)	0.0424 (11)	
H11	0.1282	−0.0021	−0.1274	0.051*	
C12	0.4193 (7)	0.0238 (5)	−0.0901 (5)	0.0547 (13)	
H12A	0.5038	0.0811	−0.0764	0.082*	
H12B	0.4603	−0.0136	−0.1565	0.082*	
H12C	0.4276	−0.0181	−0.0210	0.082*	
C13	0.1697 (9)	0.1210 (4)	−0.2284 (5)	0.0470 (13)	
C14	0.3192 (9)	0.1652 (5)	−0.2867 (6)	0.0695 (17)	
H14	0.4491	0.1614	−0.2547	0.083*	
C15	0.2742 (13)	0.2154 (6)	−0.3935 (7)	0.089 (2)	
H15	0.3748	0.2438	−0.4336	0.107*	
C16	0.0841 (14)	0.2229 (6)	−0.4391 (6)	0.085 (2)	
H16	0.0558	0.2555	−0.5111	0.102*	
C17	−0.0640 (12)	0.1837 (6)	−0.3811 (6)	0.083 (2)	
H17	−0.1939	0.1909	−0.4123	0.100*	
C18	−0.0238 (9)	0.1330 (5)	−0.2762 (5)	0.0640 (15)	
H18	−0.1269	0.1063	−0.2368	0.077*	

### Atomic displacement parameters (Å^2^)

**Table:**

	*U*^11^	*U*^22^	*U*^33^	*U*^12^	*U*^13^	*U*^23^
Br1	0.0468 (2)	0.0492 (3)	0.1123 (5)	0.0021 (4)	0.0192 (2)	0.0044 (5)
N1	0.0392 (18)	0.039 (2)	0.032 (2)	−0.0024 (16)	0.0008 (15)	0.0033 (17)
C2	0.044 (2)	0.037 (2)	0.038 (3)	−0.0071 (19)	0.0080 (18)	−0.002 (2)
C3	0.040 (2)	0.043 (3)	0.033 (3)	−0.0001 (19)	0.0018 (18)	−0.002 (2)
C31	0.058 (2)	0.046 (2)	0.033 (2)	0.001 (4)	0.0015 (17)	0.008 (3)
C32	0.073 (3)	0.069 (4)	0.053 (3)	−0.016 (4)	−0.014 (2)	−0.006 (4)
C33	0.112 (6)	0.082 (5)	0.060 (4)	−0.023 (5)	−0.027 (4)	0.003 (4)
C34	0.157 (9)	0.076 (5)	0.040 (4)	−0.049 (6)	0.001 (4)	−0.010 (4)
C35	0.142 (7)	0.052 (4)	0.059 (4)	−0.005 (4)	0.037 (4)	−0.009 (3)
C36	0.074 (3)	0.055 (3)	0.048 (3)	−0.006 (3)	0.014 (3)	−0.001 (3)
C4	0.046 (2)	0.042 (3)	0.034 (3)	0.005 (2)	0.0054 (19)	0.005 (2)
C5	0.063 (3)	0.057 (3)	0.044 (3)	0.006 (3)	0.007 (2)	0.007 (3)
O5	0.067 (3)	0.144 (5)	0.081 (3)	0.003 (3)	−0.015 (2)	0.061 (3)
C6	0.083 (4)	0.074 (4)	0.054 (3)	−0.007 (4)	0.005 (3)	0.025 (4)
C7	0.062 (3)	0.058 (3)	0.063 (3)	0.001 (3)	0.025 (3)	0.014 (3)
C8	0.057 (3)	0.057 (4)	0.064 (4)	−0.013 (3)	0.015 (3)	0.013 (3)
C9	0.050 (2)	0.052 (3)	0.047 (3)	−0.015 (2)	0.001 (2)	0.008 (2)
C10	0.041 (2)	0.043 (3)	0.036 (3)	0.000 (2)	0.0045 (19)	0.006 (2)
C11	0.051 (3)	0.041 (3)	0.037 (3)	−0.006 (2)	0.010 (2)	−0.008 (2)
C12	0.056 (3)	0.053 (3)	0.056 (3)	0.010 (2)	0.012 (2)	0.001 (3)
C13	0.062 (3)	0.042 (3)	0.038 (3)	−0.004 (2)	0.008 (2)	0.000 (3)
C14	0.069 (4)	0.074 (4)	0.067 (4)	0.010 (3)	0.021 (3)	0.017 (4)
C15	0.120 (6)	0.084 (5)	0.069 (5)	0.011 (4)	0.046 (4)	0.029 (4)
C16	0.135 (7)	0.080 (5)	0.040 (4)	0.023 (5)	0.008 (4)	0.006 (3)
C17	0.109 (5)	0.095 (5)	0.044 (4)	0.004 (5)	−0.011 (4)	0.006 (4)
C18	0.071 (4)	0.076 (4)	0.044 (3)	−0.009 (3)	−0.004 (3)	0.003 (3)

### Geometric parameters (Å, º)

**Table:**

N1—C2	1.515 (6)	C6—H6B	0.9700
N1—C10	1.525 (6)	C7—C8	1.521 (9)
N1—C11	1.530 (6)	C7—H7A	0.9700
N1—H1	0.9100	C7—H7B	0.9700
C2—C3	1.543 (5)	C8—C9	1.534 (8)
C2—H2A	0.9700	C8—H8A	0.9700
C2—H2B	0.9700	C8—H8B	0.9700
C3—C31	1.484 (5)	C9—C10	1.514 (6)
C3—C4	1.559 (5)	C9—H9A	0.9700
C3—H3	0.9800	C9—H9B	0.9700
C31—C36	1.360 (6)	C10—H10	0.9800
C31—C32	1.393 (4)	C11—C12	1.511 (7)
C32—C33	1.380 (9)	C11—C13	1.522 (8)
C32—H32	0.9300	C11—H11	0.9800
C33—C34	1.325 (12)	C12—H12A	0.9600
C33—H33	0.9300	C12—H12B	0.9600
C34—C35	1.394 (11)	C12—H12C	0.9600
C34—H34	0.9300	C13—C14	1.385 (8)
C35—C36	1.397 (9)	C13—C18	1.392 (8)
C35—H35	0.9300	C14—C15	1.393 (9)
C36—H36	0.9300	C14—H14	0.9300
C4—C5	1.528 (7)	C15—C16	1.358 (11)
C4—C10	1.535 (7)	C15—H15	0.9300
C4—H4	0.9800	C16—C17	1.350 (11)
C5—O5	1.195 (7)	C16—H16	0.9300
C5—C6	1.493 (8)	C17—C18	1.375 (9)
C6—C7	1.514 (8)	C17—H17	0.9300
C6—H6A	0.9700	C18—H18	0.9300
			
C2—N1—C10	109.3 (3)	C8—C7—H7A	108.4
C2—N1—C11	114.8 (3)	C6—C7—H7B	108.4
C10—N1—C11	112.1 (4)	C8—C7—H7B	108.4
C2—N1—H1	106.7	H7A—C7—H7B	107.5
C10—N1—H1	106.7	C7—C8—C9	115.0 (5)
C11—N1—H1	106.7	C7—C8—H8A	108.5
N1—C2—C3	106.2 (3)	C9—C8—H8A	108.5
N1—C2—H2A	110.5	C7—C8—H8B	108.5
C3—C2—H2A	110.5	C9—C8—H8B	108.5
N1—C2—H2B	110.5	H8A—C8—H8B	107.5
C3—C2—H2B	110.5	C10—C9—C8	114.4 (5)
H2A—C2—H2B	108.7	C10—C9—H9A	108.7
C31—C3—C2	114.1 (2)	C8—C9—H9A	108.7
C31—C3—C4	112.8 (2)	C10—C9—H9B	108.7
C2—C3—C4	101.5 (3)	C8—C9—H9B	108.7
C31—C3—H3	109.4	H9A—C9—H9B	107.6
C2—C3—H3	109.4	C9—C10—N1	110.4 (4)
C4—C3—H3	109.4	C9—C10—C4	115.9 (4)
C36—C31—C32	118.0 (3)	N1—C10—C4	103.0 (4)
C36—C31—C3	124.1 (3)	C9—C10—H10	109.1
C32—C31—C3	117.9 (3)	N1—C10—H10	109.1
C33—C32—C31	120.2 (4)	C4—C10—H10	109.1
C33—C32—H32	119.9	C12—C11—C13	115.7 (4)
C31—C32—H32	119.9	C12—C11—N1	111.3 (4)
C34—C33—C32	122.1 (7)	C13—C11—N1	109.7 (4)
C34—C33—H33	119.0	C12—C11—H11	106.5
C32—C33—H33	119.0	C13—C11—H11	106.5
C33—C34—C35	119.1 (7)	N1—C11—H11	106.5
C33—C34—H34	120.4	C11—C12—H12A	109.5
C35—C34—H34	120.4	C11—C12—H12B	109.5
C34—C35—C36	119.4 (7)	H12A—C12—H12B	109.5
C34—C35—H35	120.3	C11—C12—H12C	109.5
C36—C35—H35	120.3	H12A—C12—H12C	109.5
C31—C36—C35	121.1 (6)	H12B—C12—H12C	109.5
C31—C36—H36	119.4	C14—C13—C18	118.2 (5)
C35—C36—H36	119.4	C14—C13—C11	122.7 (5)
C5—C4—C10	110.6 (4)	C18—C13—C11	119.0 (5)
C5—C4—C3	112.8 (4)	C13—C14—C15	119.8 (6)
C10—C4—C3	105.3 (3)	C13—C14—H14	120.1
C5—C4—H4	109.4	C15—C14—H14	120.1
C10—C4—H4	109.4	C16—C15—C14	120.2 (6)
C3—C4—H4	109.4	C16—C15—H15	119.9
O5—C5—C6	121.2 (5)	C14—C15—H15	119.9
O5—C5—C4	119.5 (5)	C17—C16—C15	120.7 (7)
C6—C5—C4	119.4 (5)	C17—C16—H16	119.6
C5—C6—C7	118.7 (5)	C15—C16—H16	119.6
C5—C6—H6A	107.7	C16—C17—C18	120.3 (7)
C7—C6—H6A	107.7	C16—C17—H17	119.9
C5—C6—H6B	107.7	C18—C17—H17	119.9
C7—C6—H6B	107.7	C17—C18—C13	120.7 (6)
H6A—C6—H6B	107.1	C17—C18—H18	119.7
C6—C7—C8	115.4 (5)	C13—C18—H18	119.7
C6—C7—H7A	108.4		
			
C10—N1—C2—C3	9.1 (4)	C7—C8—C9—C10	61.4 (7)
C11—N1—C2—C3	−117.9 (4)	C8—C9—C10—N1	−179.8 (5)
N1—C2—C3—C31	−150.5 (2)	C8—C9—C10—C4	−63.3 (6)
N1—C2—C3—C4	−28.9 (4)	C2—N1—C10—C9	139.4 (4)
C2—C3—C31—C36	63.8 (4)	C11—N1—C10—C9	−92.1 (5)
C4—C3—C31—C36	−51.3 (4)	C2—N1—C10—C4	15.1 (4)
C2—C3—C31—C32	−118.9 (2)	C11—N1—C10—C4	143.5 (4)
C4—C3—C31—C32	125.9 (2)	C5—C4—C10—C9	83.8 (5)
C36—C31—C32—C33	0.3 (4)	C3—C4—C10—C9	−154.1 (4)
C3—C31—C32—C33	−177.1 (4)	C5—C4—C10—N1	−155.5 (4)
C31—C32—C33—C34	0.9 (9)	C3—C4—C10—N1	−33.5 (4)
C32—C33—C34—C35	−1.1 (11)	C2—N1—C11—C12	54.3 (5)
C33—C34—C35—C36	0.1 (10)	C10—N1—C11—C12	−71.1 (5)
C32—C31—C36—C35	−1.3 (6)	C2—N1—C11—C13	−75.0 (5)
C3—C31—C36—C35	176.0 (4)	C10—N1—C11—C13	159.5 (4)
C34—C35—C36—C31	1.1 (9)	C12—C11—C13—C14	−15.9 (8)
C31—C3—C4—C5	−78.0 (4)	N1—C11—C13—C14	111.1 (6)
C2—C3—C4—C5	159.5 (4)	C12—C11—C13—C18	162.8 (5)
C31—C3—C4—C10	161.3 (2)	N1—C11—C13—C18	−70.3 (6)
C2—C3—C4—C10	38.8 (4)	C18—C13—C14—C15	−3.3 (10)
C10—C4—C5—O5	109.6 (6)	C11—C13—C14—C15	175.4 (6)
C3—C4—C5—O5	−8.0 (8)	C13—C14—C15—C16	1.4 (11)
C10—C4—C5—C6	−70.3 (6)	C14—C15—C16—C17	1.2 (12)
C3—C4—C5—C6	172.1 (5)	C15—C16—C17—C18	−1.8 (12)
O5—C5—C6—C7	−172.8 (7)	C16—C17—C18—C13	−0.2 (11)
C4—C5—C6—C7	7.1 (9)	C14—C13—C18—C17	2.7 (10)
C5—C6—C7—C8	62.0 (8)	C11—C13—C18—C17	−176.0 (6)
C6—C7—C8—C9	−81.5 (7)		

### Hydrogen-bond geometry (Å, º)

**Table:**

*D*—H···*A*	*D*—H	H···*A*	*D*···*A*	*D*—H···*A*
N1—H1···Br1	0.91	2.44	3.266 (4)	151
